# Buffalo Medical and Surgical Association

**Published:** 1885-08

**Authors:** 


### Society Reports.

### Buffalo Medical and Surgical Association.

*Meeting of June 2d, 1885.*

The President, Dr. Charles G. Stockton, in the chair; Dr. Frank H. Potter, Secretary.

The minutes of last meeting were read and approved.

Dr. W. W. Potter read a paper entitled " Pelvic Abscess." This paper appeared in full in the July number of the *Buffalo Medical and Surgical Journal.*

Dr. Cronyn opened the discussion, saying the diagnosis in these cases, between pus and serum, was often difficult. This difficulty was increased when the case was not seen in its early stages. He considered that the serous was the primary stage of the formation of pus, and related a case in support of this view; also, related a case in which the diagnosis was only made by placing the patient in the genu-pectoral posture. On attempting to introduce the finger into the rectum, it passed suddenly through its wall into a large abscess cavity. He thought it a good plan to open the abscess through the rectum, as the danger was very slight in so doing.

Dr. W. S. Tremaine called attention to the fact that pelvic abscess also occurred in the male, and reported a case. He did not agree with the essayist in his views about the thermo-cautery. The latter was safer than the knife, because it lessened the power of infection. By its use, the edges of the wound were cauterized and thus prevented from absorbing any infectious material. He also believed that the delay in the closure of the wound, resulting from this condition, was of benefit, because it gave ample opportunity for treating properly the abscess cavity.

Dr. F. W. Bartlett questioned the advisability of washing out a pelvic abscess with antiseptic solutions. He would confine his treatment to keeping the parts around the wound clean, and leave the abscess to close in its natural way. It is often a good plan to plug the opening of the abscess and allow it to discharge only at regular intervals.

Dr. Hayd did not agree with Dr. Cronyn that serum was the primary stage of the formation of pus. He believed with Prof. Brickell that there were two kinds of inflammation—the phlegmenous and serous. The former ended in the formation of pus; the latter of serum. They were distinct processes, the one not resulting in the other. Reported a case of pelvic abscess occurring some weeks after labor, which was very extensive, reaching nearly to the umbilicus. There were chills, but aspiration failed to detect pus. Recovery finally took place under the use of 1-16 gr. of bichloride of mercury three times a day.

Dr. Mann said the proper treatment was immediate evacuation upon the discovery of pus. He reported some interesting cases illustrating the various channels through which the pus from an abscess might discharge. The opening into the intestines and into the bladder were both serious complications. When the opening was high up into the rectum, it seldom healed. In these cases he was in favor of making a counter opening through the vagina. He always preferred the vaginal route when possible for opening an abscess. Aspiration, for purposes of diagnosis,was not dangerous, and should be used more frequently. As an injection, he uses an iodoform emulsion with glycerine, the latter being of greater specific gravity, falls to the bottom of the cavity, the pus floating on the top, thus keeping the abscess perfectly pure.

Dr. Putnam reported a case of pelvic abscess, which opened into the rectum, and in which there was great difficulty in getting the recurrent flow in washing it out.

Dr. Clark advised reversing the aspirator and drawing the fluid out in that way, in a case like Dr. Putnam's. He advocated the more frequent use of the aspirator for diagnostic purposes.

Dr. Wetmore asked to what extent the cotton root could be considered a causative agent in the production of pelvic abscess. He had seen some cases which he believed were due to the use of this drug. It was not infrequently used to produce abortion.

Dr. Hartwig doubted this action of the cotton root. He reported a case of pelvic abscess, occurring in a pregnant woman, the evacuation of the abscess being followed by abortion. He also reported a case following gonorrhœal infection, which was complicated with peritonitis, and in which the abscess opened into the intestines.

Dr. Stockton asked the proper procedure when the abscess occurred high in the pelvis, and opened by a long and tortuous route into the rectum.

Dr. Potter, replying, said, in the last case he would advise laparotomy, and the placing the patient on the abdomen to obtain drainage. He believed it better to avoid opening the abscess into the rectum, as besides the difficulty in obtaining proper drainage, there was the liability of the entrance of fecal matters into the abscess. He did not condemn the thermo-cautery entirely, but only its use in acute abscesses. It was, perhaps, the best instrument to use in making a counter opening in order to change the route of the abscess.

He did not consider it very important to diagnose between serum and pus, as the proper treatment was evacuation in either case. He also believed in washing out the abscess cavity frequently and thoroughly with antiseptic solutions.

Dr. Grosvenor presented a specimen of intra-capusular fracture of the neck of the thigh bone. The fracture had never united, and the patient lived nine years after the accident.

The prevailing diseases reported were, cerebro-spinal meningitis, measles and whooping cough.

The Treasurer presented the following report:

In accordance with the By-Laws, I hereby prefer charges against the following members for non-payment of dues: Drs. Slacer, 8; Haberstro, 5; Smith, 6; Kamerling, 5; Earle, 8; Trowbridge, 5. (Signed,)

F. E. L. Brecht.

On motion, the President appointed the following committee to investigate these charges, and report at the next meeting: Drs. Coakley, Putnam, Clark, Fowler and F. H. Potter.

As there was no record of the election of the Secretary as a member of the Association, the minutes, on motion, were so amended as to make him a member since the 1st of January, 1885.

The Society then adjourned.

